# A public health approach to pediatric hearing impairment in the Pacific Islands

**DOI:** 10.7189/jogh.08.010302

**Published:** 2018-06

**Authors:** Annette Kaspar, Joseph Kei, Carlie Driscoll, De Wet Swanepoel, Helen Goulios

**Affiliations:** 1 ENT Clinic, National Referral Hospital, Honiara, Solomon Islands; 2 Hearing Research Unit for Children, School of Health and Rehabilitation Sciences, University of Queensland, Brisbane, Australia; 3 Department of Speech-Language Pathology and Audiology, University of Pretoria, Pretoria, South Africa; 4 School of Anatomy, Physiology and Human Biology, University of Western Australia, Perth, Australia

Childhood hearing impairment is a significant cause of disability in developing countries, where infectious diseases are the leading cause of avoidable pediatric hearing loss [1]. The World Health Organization (WHO) theme for World Hearing Day 2016 was “Childhood hearing loss: act now, here’s how!”, with a focus on preventable childhood hearing impairment through public health measures [1]. This message was reprised for World Hearing Day 2017 with “Action for hearing loss: make a sound investment”. The integration of pediatric audiology services with public health initiatives, combined with sustainable capacity-development and training of local health professionals, should reduce the burden of pediatric hearing impairment in developing countries, and make a positive contribution to the United Nations Sustainable Development Goals (SDGs). The WHO defines public health as “the art and science of preventing disease, prolonging life and promoting health through the organized efforts of society”.

The provision of hearing services in the Pacific Islands is currently limited [2]. Although there is very little in the audiology literature on hearing disorders in Pacific nations, this region is estimated to have among the world’s highest burden of hearing impairment due to meningitis and otitis media (OM) [3]. Childhood immunization coverage rates remain below target levels [4], and early-onset otitis media is more likely to progress to chronic and fatal complications [3]. A detailed review of the data on the prevalence of childhood hearing loss in the Pacific Islands and their etiologies (ie, malaria/measles/mumps/ototoxicity) is provided elsewhere in the literature [3].

The WHO reports that 60% of global childhood hearing loss is preventable: prenatal and postnatal infections (31%), birth-related causes (17%), ototoxic medicines (4%), and other causes such as substance abuse (8%) [1]. A public health approach to the development of pediatric audiology services should address these major causes of preventable hearing loss in the Pacific Islands, and the WHO and the United Nations Children’s Emergency Fund (UNICEF) are involved in a number of initiatives that could offer a platform for this model of childhood hearing service delivery.

## INTEGRATION OF AUDIOLOGY SERVICES WITH EXISTING PUBLIC HEALTH PROGRAMS

The integration of new pediatric audiology services into existing public health care programs should have a positive outcome if these programs are accepted and well-attended by the community. Given the success of the WHO Expanded Program on Immunization in developing countries, immunization clinics have been identified as an alternative platform for Infant Hearing Screening (IHS) programs in nations where a hospital-based Universal Newborn Hearing Screening Program may not be feasible [5]. The current WHO and UNICEF agendas for the Pacific Islands continue to prioritize routine childhood immunizations [6,7], and the integration of IHS with this important public health service is an attractive possibility. Even if the hearing screening assessment involves a questionnaire rather than more sophisticated methods such as Oto-Acoustic Emissions (OAEs) or Automated Auditory Brainstem Response (AABR), IHS at immunization clinics offers an opportunity to (1) perform an ear health check for a population that is at greater risk of early-onset otitis media, and (2) provide caregiver education on ear and hearing health. While all WHO member states should aim to identify and provide intervention for infants with sensorineural (ie, permanent) hearing loss before they reach 12 months of age, this is unlikely to be feasible for Pacific Island countries in the near future. The implementation of a basic ear and hearing health program is an interim measure that should establish the infrastructure in anticipation of more sophisticated programs. A successful basic program would be more likely to attract the support of the WHO World Wide Hearing Project which adheres to the principle of program sustainability.

School-screening programs are another well-known public health approach to managing significant health conditions and improving educational outcomes. The development of school-based ear and hearing assessments in the Pacific Islands may collaborate with the WHO Global School Health Initiative to optimize successful implementation of the program [8]. This initiative also presents a platform for health promotion activities where one public health message (eg, nutrition) may target numerous health conditions, including otitis media and associated hearing impairment. The Initiative also promotes collaboration between the Ministries of Health and Education to achieve their key targets of the Sustainable Development Goals.

**Figure:**
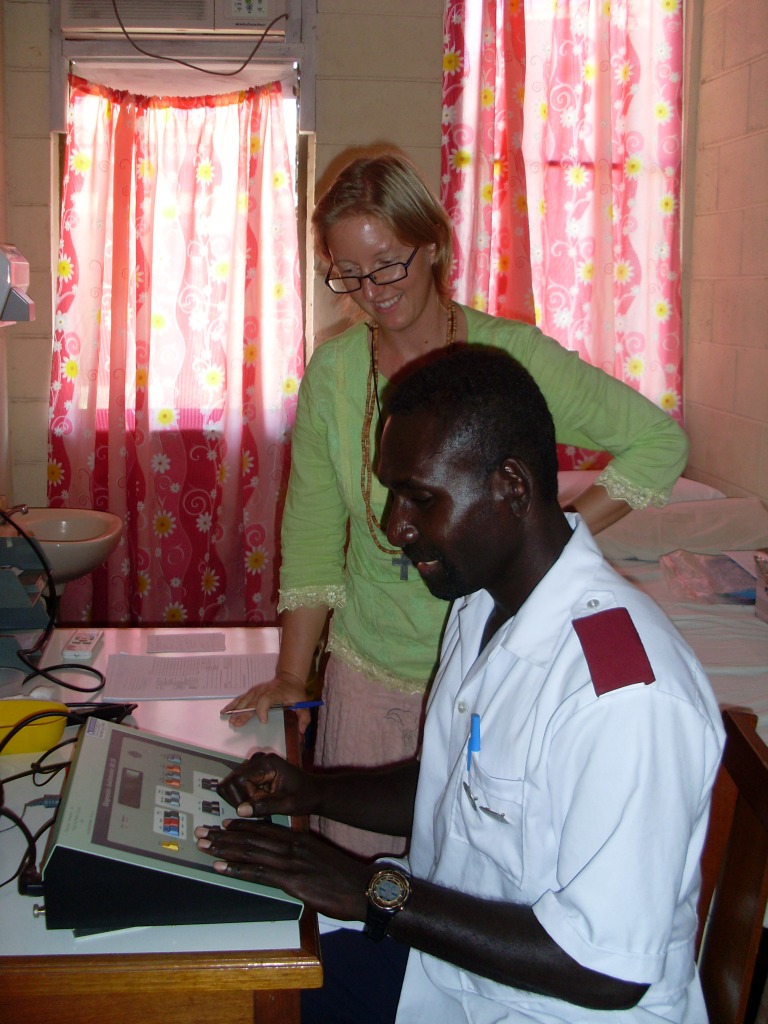
Photo: ENT Nurse-Audiometrist Obiga Newton (Solomon Islands) working with Audiologist Annette Kaspar (Australia). From the collection of Annette Kaspar (used with permission).

There are a number of challenges (ie, transport) that may prevent attendance at a hospital or health clinic. School hearing screening programs offer an acceptable community-based alternative, and would enable early access to primary health care services for ear/hearing disorders. A key aim of these programs would be to facilitate medical and, possibly, rehabilitative and educational interventions for children identified with a hearing impairment.

## DEVELOPMENT OF PUBLIC HEALTH AUDIOLOGY SERVICES IN COLLABORATION WITH LOCAL ENT CLINICS

Hospital-based Ear, Nose & Throat (ENT) Clinics are among the best candidates for the development of public health hearing services for children in the Pacific Islands. Given their existing commitment to otological health care, ENT Clinic staff members are the most likely to appreciate the additional contribution of pediatric audiology services in the overall diagnosis and management of a child with OM-induced hearing loss. ENT Clinic staff members may also have the greatest motivation for acquiring audiometry skills, satisfying the principle of capacity-development in the implementation of locally sustainable services. An opportunity for formal qualifications would be desirable as it provides local staff members with recognition for their skills.

Where ENT medical services are accepted and well-attended by the community, the integration of audiometry services into the ear health care framework has been successful. At the time of writing, ENT Registered Nurses in the Solomon Islands and Vanuatu are able to provide audiometry testing as part of their ENT outpatient service. Given that these ENT nurses include a school outreach service as part of their clinical program, the development of an ear and hearing screening program performed by the ENT nurses is a clear possibility. The ENT Clinic may also extend their community-based services to Infant Ear and Hearing Screening programs, targeting early-onset otitis media, and monitoring infants at risk of sensorineural hearing impairment.

In addition to community-based public health programs, a hospital-based audiology service should consider the implementation of a hospital-based hearing screening program for survivors of meningitis. Given the estimated burden of post-meningitic hearing impairment in the Pacific Region, the results of a routine hearing screening program for all meningitis survivors could lend extra support to the implementation of immunizations against the multiple causal pathogens of meningitis. A hospital-based hearing screening program may eventually expand to assess other patients at risk of hearing loss (ie, ototoxicity).

## (RE)HABILITATION FOR CHILDREN WITH PERMANENT HEARING IMPAIRMENT IN THE PACIFIC ISLANDS

Although the focus of the present review has been a public health approach to reducing the burden of preventable and treatable causes of childhood hearing impairment in the Pacific Islands, some comments about (re)habilitation audiology services are warranted as pilot Infant Hearing Screening programs will identify children with permanent congenital and early-onset hearing loss. In accordance with the principles of international health development, the implementation of (re)habilitation audiology services must consider sustainability. Indeed, the WHO recommends the provision of amplification devices (eg, hearing aids, cochlear implants) only where support and maintenance services are also available [9]. The additional challenges of hearing aid maintenance and management due to high heat and humidity in the Pacific Islands must be considered.

Although many Pacific Island nations have a School for the Deaf or special schools for children with disabilities, the provision of hearing aids for students is limited [2]. While there are arguments that early identification programs should not be implemented if (re)habilitation services are unavailable, diagnosis alone of a permanent hearing impairment should be a positive outcome for children whose condition may otherwise be attributed to an intellectually disability or a non-biomedical/cultural cause (eg, spiritual attack, witchcraft). An early diagnosis of a permanent hearing impairment allows caregivers to make decisions about the long-term options for their child, such as enrolment in a School for the Deaf or School for Children with a Disability. The ethical considerations associated with providing a pediatric diagnostic audiology service in a resource-limited setting are presented elsewhere in the literature [10].

Should the provision of rehabilitation audiology services be sustainable, there may be more community acceptance of hearing aid services if they are delivered at a School for the Deaf or special school, since a hospital-based service may inadvertently lead to negative perceptions of permanent hearing loss primarily as a disease or illness. Referral pathways should, however, continue to exist between such centres and the ENT Clinic to ensure any additional ear disease is managed as required. Given the tropical climate of many Pacific Island nations, the use of hearing aids increases the risk of otitis externa, as well as cerumen impaction.

Another benefit of a school-based service would be that hearing aid care and maintenance are provided by staff in regular contact with the hearing-impaired child. It must be noted, however, that the Pacific Island region is currently moving towards Inclusive Education strategies rather than encouraging separate schools for children with a disability. The Inclusive Education policies are largely based on the fact that attendance at special schools is difficult, and that Inclusive Education is more appropriate given the economic and geographic challenges of the Pacific Island context.

## CONCLUSION

The development of pediatric audiology services in the Pacific Islands should consider a public health approach. The integration of childhood hearing services with existing public health initiatives by international organizations such as the WHO and UNICEF, should reduce the burden of preventable childhood hearing loss in the Pacific Islands. Collaboration with the local ENT Clinic, including training and capacity-development of ENT Clinic staff members, is an attractive option in the development of local audiology services for children.
